# Supplementary material for: Bacterial diversity in arboreal ant nesting spaces is linked to colony developmental stage
Source: Commun Biol. 2023 Nov 30;6:1217. doi: 10.1038/s42003-023-05577-5 (PMC10689775; doi:10.1038/s42003-023-05577-5)
Supplement: Supplementary file 2 — Supplementary Information [file 42003_2023_5577_MOESM2_ESM.pdf]

Supplementary Information for:

Bacterial diversity in arboreal ant nesting spaces is linked to colony developmental stage

Maximilian Nepel, Veronika E. Mayer, Veronica Barrajon-Santos and Dagmar Woebken

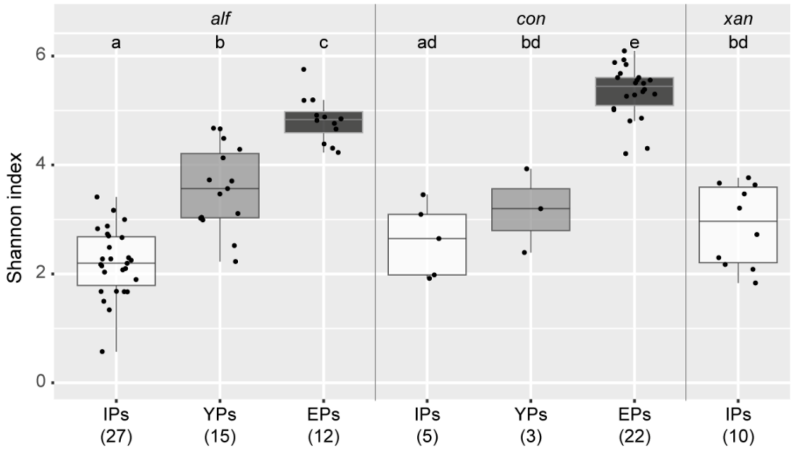

Supplementary Figure 1: Shannon indices, depicting the alpha diversity in patches of initial (IP), young (YP) and established (EP) ant colonies separated by the *Azteca* ant species (alf: *A. alfari*, con: *A. constructor*, xan: *A. xanthochroa*). Sampling sizes are given in brackets. Letters denote significant differences between patch types identified using pairwise Wilcoxon rank sum test. Please note that an average bacterial community was calculated per spatially multiple sampled established ant colony.

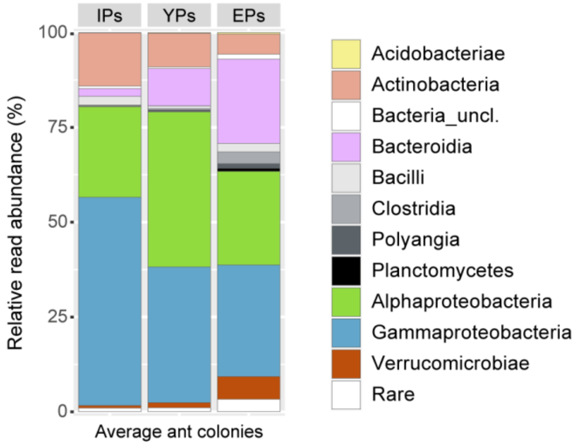

Supplementary Figure 2: Bacterial community composition along developmental stages of ant colonies, including patches of initial (IP), young (YP) and established (EP) ant colonies. Taxonomic bar plot depicting the relative read abundance of classes on the y-axis. On the x-axis, every bar represents the average bacterial community composition per ant colony developmental stage. Please note that displaying average community compositions per ant colony developmental stage is a simplification due to the heterogeneity across patch samples (see Figure 2a, 3a, 5a).

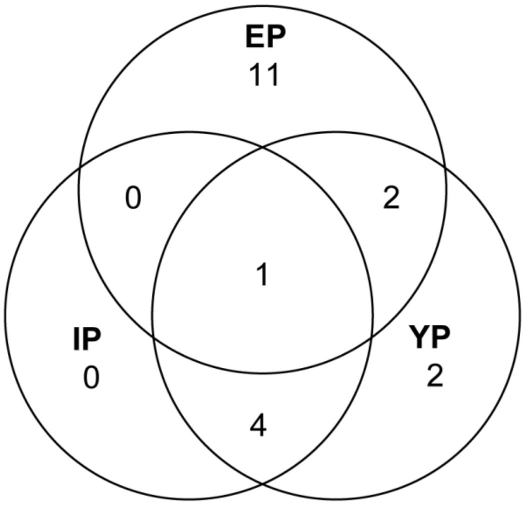

Supplementary Figure 3: Venn diagrams depicting the distribution of prevalent ASVs across patches of initial (IP), young (YP) and established (EP) ant colonies. ASVs were defined as prevalent if they accounted for at least 0.4% of reads in more than 50% of either IP, YC, or EP (EP_alf, EP_con) samples. The number of ASVs prevalent in more than one patch type is visible in respective intersections.

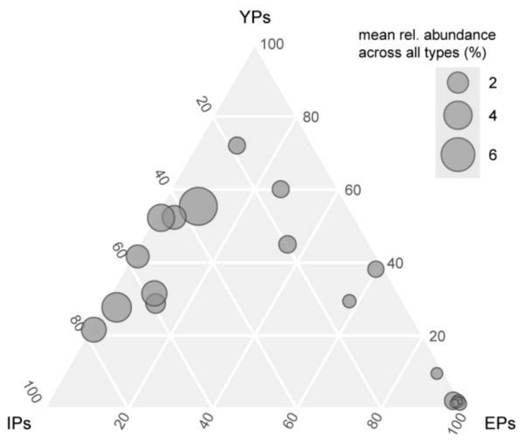

Supplementary Figure 4: Ternary plots depicting the distribution of prevalent ASVs across ant colonies' developmental stages (initial (IP), young (YP) and established (EP) ant colonies). ASVs were defined as prevalent if they accounted for at least 0.4% of reads in more than 50% of either IP, YP, or EP samples. Each circle represents one ASV. The position of the circle in the ternary plot depicts the ratio of reads of this ASV in the different developmental stages. The circle size depicts the mean relative abundance across all patch types.

Supplementary Table 1: Mean relative read abundance (%) of selected orders in patches of established *A. alfari* and *A. constructor* colonies. Asterisks denote statistically significant changes in relative read abundances between both ant species (** < 0.01, * < 0.05)

		**Established ant colonies**		
**Selected orders**	**Class**	***A. alfari***	***A. constructor***	
Corynebacteriales	Actinobacteria	1.9	1.1	*
Frankiales	Actinobacteria	0.4	0.6	
Micrococcales	Actinobacteria	1.7	2.6	
Bacteroidales	Bacteroidia	0.4	2.4	**
Chitinophagales	Bacteroidia	9.8	13.9	
Cytophagales	Bacteroidia	1.6	2.1	
Flavobacteriales	Bacteroidia	3.7	5.9	
Sphingobacteriales	Bacteroidia	1.3	2.3	
Bacillales	Bacilli	0.8	1.3	
Lactobacillales	Bacilli	0.1	2.0	*
Lachnospirales	Clostridia	1.2	3.1	*
Polyangiales	Myxococcia	0.6	1.3	
Caulobacterales	Polyangia	1.9	1.4	
Rhizobiales	Alphaproteobacteria	16.1	8.0	**
Rhodobacterales	Alphaproteobacteria	4.7	4.2	
Sphingomonadales	Alphaproteobacteria	5.1	5.8	
Burkholderiales	Alphaproteobacteria	19.6	16.6	
Enterobacterales	Gammaproteobacteria	1.5	2.0	
Pseudomonadales	Gammaproteobacteria	3.1	2.8	
Xanthomonadales	Gammaproteobacteria	6.6	4.0	*
Chthoniobacterales	Gammaproteobacteria	0.7	0.8	
Verrucomicrobiales	Verrucomicrobiae	7.7	2.2	*

Supplementary Table 2: Measurement of carbon and nitrogen content in *Cecropia* parenchyma commonly used by *Azteca* queens to form patches in early ant colony stages. Parenchyma tissue was sampled inside uncolonized plant internodes. C/N ratio was calculated subsequently.

Sample ID	Sample weight [mg]	N [Amt%]	C [Amt%]	C/N ratio
CR15Cec01Par	1.277	0.15	34.31	221.39
CR15Cec02Par	1.131	0.18	55.12	303.87
CR15Cec07Par	1.135	0.39	40.42	102.96
CR15Cec21Par	1.625	0.20	21.98	108.40
CR15Cec04Par	1.498	0.28	45.08	163.86
